# Serum S100B protein as a marker of severity in Covid-19 patients

**DOI:** 10.1038/s41598-020-75618-0

**Published:** 2020-10-29

**Authors:** Antonio Aceti, Lory Marika Margarucci, Elena Scaramucci, Massimiliano Orsini, Gerardo Salerno, Gabriele Di Sante, Gianluca Gianfranceschi, Rosa Di Liddo, Federica Valeriani, Francesco Ria, Maurizio Simmaco, Pier Paolo Parnigotto, Matteo Vitali, Vincenzo Romano Spica, Fabrizio Michetti

**Affiliations:** 1 Sant’Andrea Hospital A.O.U., Sapienza University of Rome, Via di Grottarossa 1035, 00189 Rome, Italy; 2 Department of Movement, Human and Health Sciences, Laboratory of Epidemiology and Biotechnologies, University of Rome “Foro Italico”, Piazza Lauro De Bosis 6, 00135 Rome, Italy; 3 Istituto Zooprofilattico Sperimentale Delle Venezie, Viale dell’Università 10, 35020 Legnaro, Padua, Italy; 4 Department of Translational Medicine and Surgery, Section of General Pathology, Università Cattolica del Sacro Cuore, Largo Francesco Vito 1, 00168 Rome, Italy; 5 Fondazione Policlinico Universitario A. Gemelli - IRCCS, Largo A. Gemelli 1-8, 00168 Rome, Italy; 6 Department of Pharmaceutical and Pharmacological Sciences, University of Padua, Via Marzolo 5, 35131 Padua, Italy; 7 Foundation for Biology and Regenerative Medicine, Tissue Engineering and Signaling (T.E.S.) Onlus, Via De Sanctis 10, 35030 Caselle di Selvazzano Dentro, Padua, Italy; 8 Department of Public Health and Infectious Diseases, Sapienza University of Rome, Piazzale Aldo Moro, 5, 00185 Rome, Italy; 9 Department of Neuroscience, Università Cattolica del Sacro Cuore, Largo Francesco Vito 1, 00168 Rome, Italy; 10 IRCCS San Raffaele Scientific Institute, Università Vita-Salute San Raffaele, Via Olgettina, 58, 20132 Milan, Italy

**Keywords:** Biomarkers, Diseases, Medical research

## Abstract

SARS-CoV-2 infection shows a wide-ranging clinical severity, requiring prognostic markers. We focused on S100B, a calcium-binding protein present in biological fluids, being a reliable biomarker in disorders having inflammatory processes as common basis and RAGE as main receptor. Since Covid-19 is characterized by a potent inflammatory response also involving RAGE, we tested if S100B serum levels were related to disease severity. Serum samples (n = 74) were collected from hospitalized SARS-CoV-2 positive patients admitted to Covid center. Illness severity was established by admission clinical criteria and Covid risk score. Treatment protocols followed WHO guidelines available at the time. Circulating S100B was determined by ELISA assay. Statistical analysis used Pearson’s χ^2^ test, t-Test, and ANOVA, ANCOVA, Linear Regression. S100B was detected in serum from Covid-19 patients, significantly correlating with disease severity as shown both by the level of intensity of care (*p* < 0.006) as well by the value of Covid score (Multiple R-squared: 0.3751); the correlation between Covid-Score and S100B was 0.61 (*p* < 0.01). S100B concentration was associated with inflammation markers (Ferritin, C-Reactive Protein, Procalcitonin), and organ damage markers (Alanine Aminotransferase, Creatinine). Serum S100B plays a role in Covid-19 and can represent a marker of clinical severity in Sars-CoV-2 infected patients.

## Introduction

Evaluation of Covid-19 severity and possible outcomes is limited by clinical heterogeneity and lack of specific markers^1–3^. Several laboratory parameters are considered in clinical practice, but the identification of novel indicators in blood specimens is a key issue for understanding the underlying biological mechanisms and improving prognostic accuracy^4–9^.As a candidate marker we focused on the S100B protein, which is regarded to be involved in inflammatory processes as a Danger-Associated Molecular Patterns (DAMP) molecule^10,11^. S100B is a small acidic calcium-binding protein, originally isolated in the nervous system, where it is concentrated in astrocytes, being also present in oligodendrocytes, Schwann cells, enteric glial cells, and some neuron subpopulations^12,13^. It is also present in definite non-neural cell types, including dendritic cells, certain lymphocyte subpopulations, chondrocytes, Langerhans cells, melanocytes, adrenal medulla satellite cells, Leydig cells, skeletal muscle satellite cells, and adipocytes, which intriguingly constitute a site of concentration for the protein comparable to the nervous tissue^10^. S100B can be detected in biological fluids (cerebrospinal fluid, peripheral blood, urine, saliva, feces) in particular conditions^10,12,14^. Besides, the protein has been shown to be actively released and interact with target cells through the multiligand transmembrane immunoglobulin-like Receptor for Advanced Glycation Endproducts (RAGE) which is able to initiate an intracellular signaling cascade^15^ and was associated to several pathological conditions, reasonably referred to inflammatory processes^12^. Based on these findings, S100B is considered a reliable biomarker for a variety of neural and non-neural pathological conditions, even displaying a predictive role^14–17^.

Indeed, S100B appears to share similar characteristics to DAMPs molecules, including the interaction with RAGE and, once released in the microenvironment, the active participation to the inflammatory tissue reaction to damage^10,18^. Interestingly, the S100B-RAGE axes has been shown to participate in pulmonary inflammatory processes^19,20^.

Thus, in the light of the notion that SARS CoV-2 infection can induce a severe acute respiratory syndrome with a complex pattern of clinical manifestations characterized by a potent inflammatory response^18,21–23^ we tested the possibility that S100B could be present in detectable amounts in serum of Covid-19 patients, as well as the possible relationship between severity of the disease and increase in S100B serum levels.

## Results

### S100B is present in serum of Covid patients, correlating with disease severity

**Figure 1:**
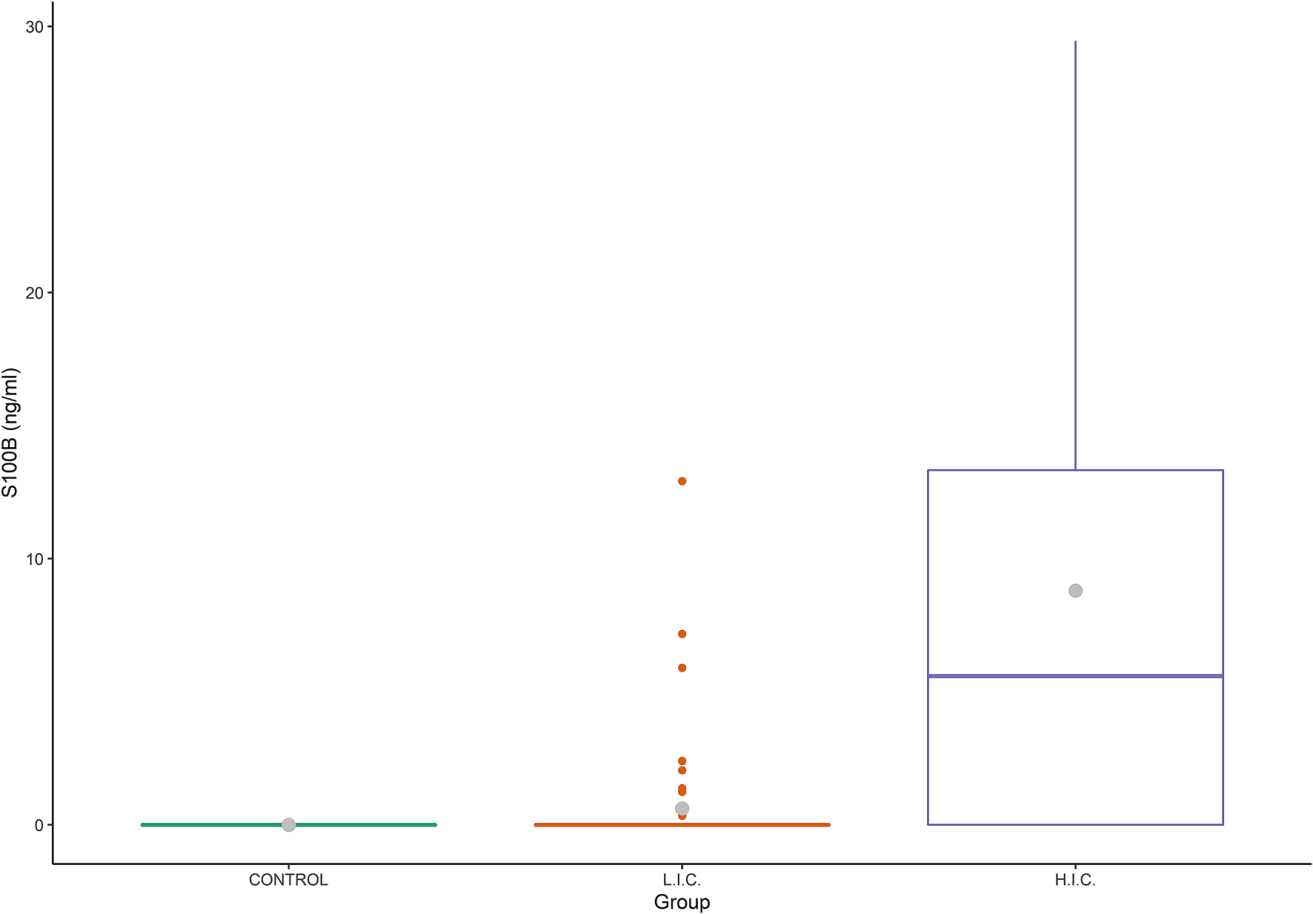
Detection of S100B in Covid patients and controls. Box plot showing the distribution of S100B in Covid (n = 74) patients with different clinical severity of disease. Also data from controls (n = 5 healthy individuals: negative for SARS-CoV-2 detection by PCR and negative by serologic test) are included. Grey dot: mean value, Line: median value; HIC: High Intensity Care; LIC: Low Intensity Care.

S100B was detected at concentrations over the LOD in the serum of 19 patients out of 74 (25.7%), ranging from 0.25 to 29.46 ng/mL. Results obtained by non-parametric Wilcox test showed a positive significant association (*p* < 0.001) between S100B serum concentrations and the severity of the disease as measured based on HIC or LIC wards where the Covid-19 patients were hospitalized. S100B levels showed a higher mean value in HIC than in LIC (8.80 ± 10.24 and 0.62 ± 2.10 ng/mL, respectively; *p* < 0.006), as reported in Table 1 and Fig. 1.

**Table 1:** Overview of patients included in the study and their serum data.

Participants	All (n = 74)	HIC^a^ (n = 19)	LIC^b^ (n = 55)	*p* value
**Characteristics**				
Median age (IQR)—years	66 (32–89)	63 (35–85)	66 (32–89)	0.66
Female – number (%)	25 (49)	9 (64)	16 (43)	
Period from hospitalization to blood sample collection—days (SD)	18.0 ± 18.0	19.5 ± 17.8	17.6 ± 18.3	0.72
Period from blood sample collection to hospital discharge—days (SD)	13.2 ± 11.5	14.0 ± 11.8	13.0 ± 11.5	0.79
**Serum data**				
S100B—ng/mL (SD)	2.39 ± 6.04	8.80 ± 10.24	0.62 ± 2.10	0.006
White Blood Cell count per mm^3^ (SD)	7.21 ± 3.01	7.78 ± 4.56	7.05 ± 2.45	0.55
Lymphocyte count per mm^3^ (SD)	1.49 ± 0.73	1.41 ± 0.85	1.51 ± 0.69	0.67
Alanine Aminotransferase—IU/L (SD)	29.8 ± 26.2	39.4 ± 43.0	27.2 ± 18.9	0.28
Creatinine—mg/dL (SD)	0.96 ± 0.57	0.89 ± 0.45	0.98 ± 0.60	0.52
d-Dimer—ng/mL (SD)	583 ± 514	810 ± 585	520 ± 479	0.08
Prothrombin—seconds (SD)	13.7 ± 2.0	13.9 ± 1.9	13.6 ± 2.1	0.62
Ferritin—mg/L (SD)	782 ± 914	1212 ± 1387	663 ± 705	0.16
Procalcitonin—ng/mL (SD)	334 ± 583	586 ± 1192	265 ± 197	0.30
C Reactive Protein—mg/dL (SD)	6.00 ± 7.60	6.27 ± 8.34	5.92 ± 7.46	0.88

^a^High Intensity Care ward.

^b^Low Intensity Care ward.

No statistically significant differences were observed for other variable and in particular among the groups for age, gender and number of days of hospitalization to the date of blood sampling. The Wilcox test showed no statistically significant differences between S100B levels and gender.

**Figure 2:**
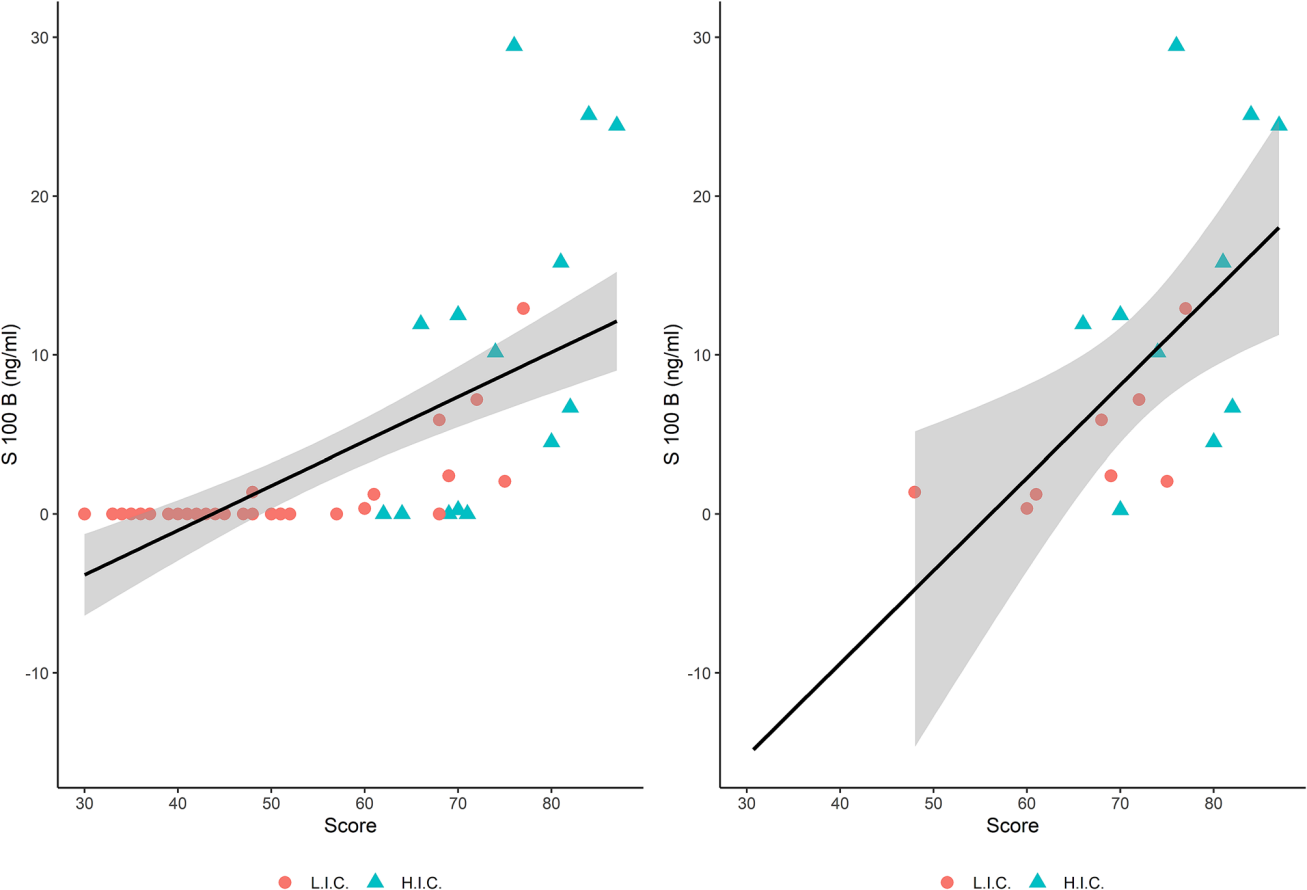
Relationship between Covid-Score and concentration of S100B. The scatterplot shows a positive correlation between S100B concentration (ng/mL) and clinical severity of the disease as represented by Covid-score. Analysis considering all samples (**A**) or only those with S100B detected in serum (**B**), from both HIC and LIC wards. Most of the samples with a concentration of S100B below the LOD belongs to the group hospitalized in the LIC wards (**A**). When considering only patients with S100B over the LOD and from both wards (**B**), the regression equation was Y = 0.584X−32.778. Figure A is reported as a comparison respect to figure B to highlight the distribution of S100B levels below the LOD and the independent linear regression curves are reported in supplementary materials (S1). The hospitalization ward is indicated for each patient (Red Dots: Low Intensity Care (LIC); Blue Triangles: High Intensive Care (HIC). The linear regression lines and their confidence intervals (95%) are showed by the gray areas. The correlation between Covid-Score and S100B is equal to 0.66 (*p* < 0.001) (**A**) and 0.61 (*p* < 0.01) (**B**).

Finally, the number of patients positive for serum S100B was significantly higher in HIC (*p* < 0.01). This result was confirmed by both Welch-ANOVA model (*p*  < 0.01) and Games-Howell test (*p* < 0.01). The incremental trend of S100B vs clinical severity measured by Covid-score was statistically significant. Figure 2 shows the distribution of S100B concentration and Covid Score in all patients (A) and the subgroup of patient where S100B was detected in the serum (B). Most patients admitted in LIC ward show a presence of S100B below the LOD, even if with different values of Covid Score (A). Additional information is reported in supplementary material (Figs. S1 and S2). When considering only those samples over the LOD, a hypothetical trendline was extrapolated (B), suggesting a theorical distribution. Both trendlines show significant correlations between S100B and the score for evaluating clinical risk (multiple R-squared 0.4369 and 0.3751, respectively), supporting a putative role of S100B as a marker for clinical severity.

### S100B correlates with several blood markers

**Figure 3:**
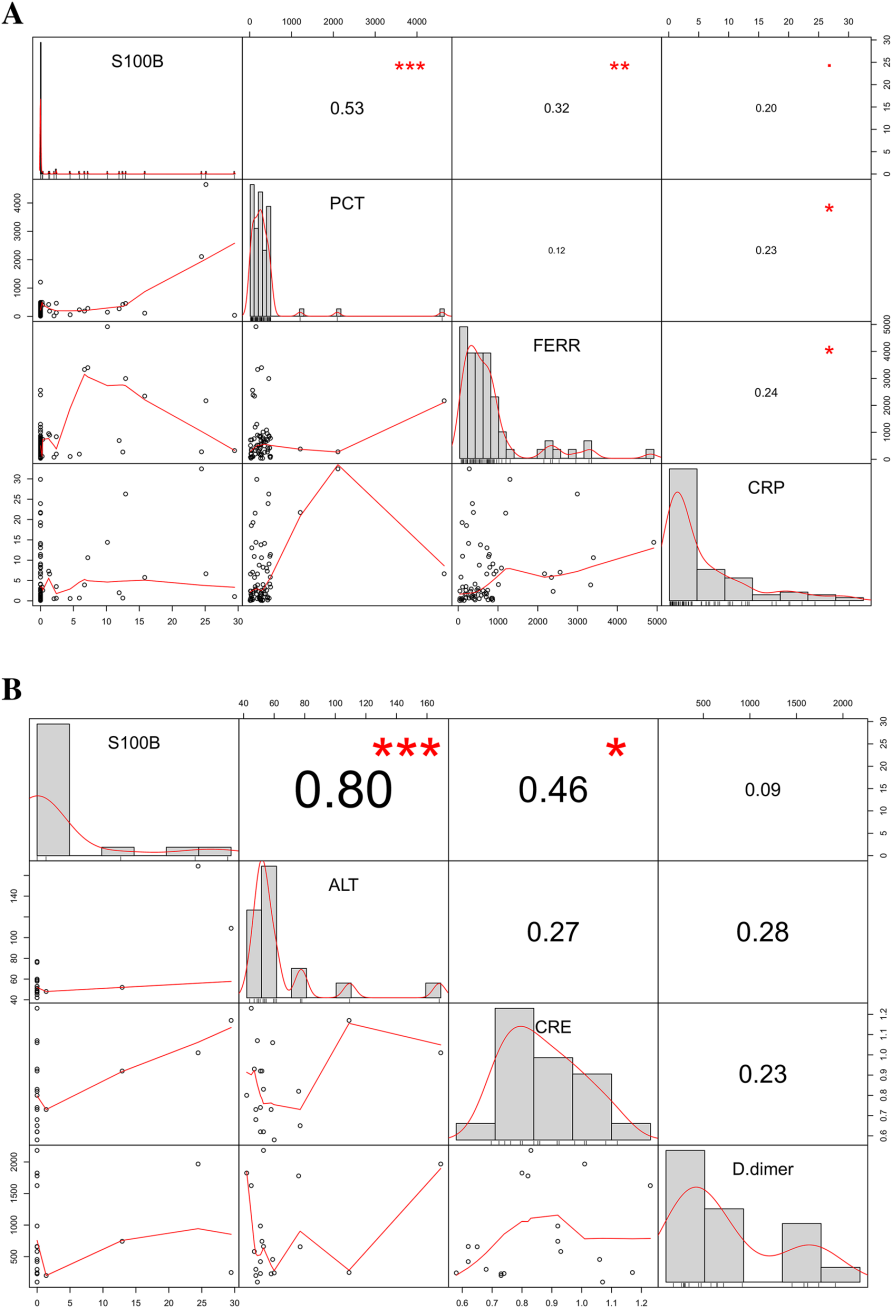
Correlation of S100B with other blood markers. (**A**) Correlation of S100B versus inflammatory markers. S100B significantly correlates with PCT, FERR, CRP. Scatterplots of pairwise variable are shown. Values in the middle of boxes are referred to the Pearson Correlation. PCT: Procalcitonin; FERR: Ferritin, CRP: C-Reactive Protein. Red Stars and dots are referred to the p-values (***p < 0.0001; **p < 0.001; *p < 0.01; ^+^*p* < 0.1). (**B**) Correlation of S100B versus Organ Damage markers. S100B significantly correlates with ALT and CRE (subgroup of patients showing ALT > 40 IU/L). Scatterplots of pairwise variable are shown. Values in the middle of boxes are referred to the Pearson Correlation. ALT: Alanine Aminotransferase; CRE: Creatinine; d-Dimer: d-Dimer. Values in the middle of boxes are referred to the Pearson Correlation. Red Stars and dots are referred to the *p* values (***p < 0.0001; **p < 0.001; *p < 0.01; ^+^*p* < 0.1).

A positive and significant correlation was observed between S100B and Ferritin concentrations (*p* < 0.01, 74 Observations), as well as for other parameters (Table 2), and in particular PCT (*p* < 0.01), d-Dimer (*p* < 0.05) and CRP (*p* < 0.1). The ANCOVA model displayed the marginal effects of the CRP and the hospitalization ward (HIC as corner point) variables on the S100B levels, showing a positive and statistically significant relation (*p* < 0.05 and *p* < 0.01, respectively). Similar results were found for PCT (*p* < 0.01) and ALT (*p* < 0.01). In order to estimate the marginal effects of the measured parameters, a stepwise selection procedure was carried out, showing ALT and PCT variables as the best predictors of the S100B levels. A further linear regression was used to verify the single and interactive effects of CRP, ALT, PCT variables, showing a significant interaction with ALT and PCT (*p* < 0.001) and a correlation with CRP (Fig. 3). The whole of the results suggests that both S100B presence and levels correlate with the severity of the disease, the trend of CRP and Ferritin values and inflammatory status, as well as with other key parameters of Covid-19 severity, such as PCT, d-Dimer, ALT. Moreover, we observed a significant correlation between S100B and CRE levels (*p* < 0.01) in the subgroup of patients with high levels of ALT (> 40 IU/L, n = 19)^18^, thus suggesting the possibility of an independent association with a liver and/or kidney damage.

**Table 2:** Correlation matrix: Pearson correlation coefficients and relative *p* values.

	S100B^a^	WBC^b^	LYM^c^	ALT^d^	CRE^e^	d-Dimer^f^	PT^g^	FERR^h^	PCT^i^	CRP^j^	age^k^	days before^l^	days after^m^	Covid score^n^
	n = 74	n = 74	n = 74	n = 74	n = 74	n = 74	n = 74	n = 69	n = 74	n = 74	n = 74	n = 74	n = 71	n = 56
S100B^a^		0.05	0.03	**0.36**	0.1	0.21	− 0.06	**0.32**	**0.53**	0.2	− 0.14	− 0.07	− 0.03	**0.66**
n = 74		0.7	0.805	0.002**	0.402	0.073^**+**^	0.599	0.007**	0.001***	0.082^+^	0.245	0.557	0.83	0.001***
WBC^b^	0.05	−	0.13	− 0.09	0.22	0.12	0.15	**0.3**	0.14	0.07	0.06	− 0.12	0.07	− 0.08
n = 74	0.7	−	0.266	0.441	0.06^+^	0.324	0.188	0.011*	0.223	0.542	0.595	0.303	0.54	0.579
LYM^c^	0.03	0.13	−	− 0.17	− 0.04	− 0.21	− 0.1	− 0.15	0.04	− **0.23**	− 0.2	− 0.11	− **0.28**	− 0.004
n = 74	0.805	0.266	−	0.138	0.717	0.067^**+**^	0.385	0.209	0.723	0.046*	0.096^+^	0.331	0.02*	0.974
ALT^d^	**0.36**	− 0.09	− 0.17	−	− 0.11	**0.32**	− 0.09	− 0.02	0.12	**0.31**	− **0.23**	− 0.13	0.03	0.11
n = 74	0.002**	0.441	0.138	−	0.347	0.006**	0.452	0.866	0.299	0.008**	0.048*	0.279	0.84	0.432
CRE^e^	0.1	0.22	− 0.04	− 0.11	−	− 0.03	0.21	− 0.13	**0.29**	− 0.05	0.2	0.02	0.03	− 0.09
n = 74	0.402	0.06^+^	0.717	0.347	−	0.822	0.073^+^	0.276	0.013*	0.701	0.09^+^	0.895	0.83	0.506
d− Dimer^f^	0.21	0.12	− 0.21	**0.32**	− 0.03	−	0.08	**0.32**	0.15	**0.26**	− 0.01	− 0.15	0.02	0.21
n = 74	0.073^+^	0.324	0.067^+^	0.006**	0.822	−	0.509	0.008**	0.197	0.026*	0.921	0.19	0.89	0.117
PT^g^	− 0.06	0.15	− 0.1	− 0.09	0.21	0.08	−	0.05	− 0.03	− 0.07	0.03	**0.23**	**0.23**	− 0.07
n = 74	0.599	0.188	0.385	0.452	0.073^+^	0.509	−	0.678	0.791	0.539	0.805	0.049*	0.05*	0.612
FERR^h^	**0.32**	**0.3**	− 0.15	− 0.02	− 0.13	**0.32**	0.05	–	0.12	**0.24**	0.09	− 0.1	− 0.04	**0.38**
n = 69	0.007**	0.011*	0.209	0.866	0.276	0.008**	0.678	–	0.333	0.043*	0.447	0.435	0.76	0.005***
PCT^i^	**0.53**	0.14	0.04	0.12	**0.29**	0.15	− 0.03	0.12	–	**0.23**	− 0.05	− 0.16	0.02	**0.28**
n = 74	0.000***	0.223	0.723	0.299	0.013*	0.197	0.791	0.333	–	0.048*	0.693	0.177	0.89	0.04*
CRP^j^	0.2	0.07	− **0.23**	**0.31**	− 0.05	**0.26**	− 0.07	**0.24**	**0.23**	–	0.07	− 0.05	0.15	0.04
n = 74	0.082^+^	0.542	0.046*	0.008***	0.701	0.026*	0.539	0.043*	0.048*	–	0.538	0.661	0.2	0.766
age^k^	− 0.14	0.06	− 0.2	− **0.23**	0.2	− 0.01	0.03	0.09	− 0.05	0.07	–	**0.26**	0.24	− 0.06
n = 74	0.245	0.595	0.096^+^	0.048	0.09^+^	0.921	0.805	0.447	0.693	0.538	–	0.026*	0.05^+^	0.63
days before^l^	− 0.07	− 0.12	− 0.11	− 0.13	0.02	− 0.15	**0.23**	− 0.1	− 0.16	− 0.05	**0.26**	–	**0.75**	0.11
n = 74	0.557	0.303	− 0.2	0.279	0.895	0.19	0.049*	0.435	0.177	0.661	0.026*	–	0.001***	0.422
days after^m^	− 0.03	0.07	**0.096**	0.03	0.03	0.02	0.23	− 0.04	0.02	0.15	0.24	**0.75**	–	0.05
n = 71	0.83	0.54	0.02*	0.84	0.83	0.89	0.05^+^	0.76	0.89	0.2	0.05^+^	0.001***	–	0.72
Covid score^n^	**0.66**	− 0.08	− 0.004	0.11	− 0.09	0.21	− 0.07	**0.38**	**0.28**	0.04	− 0.06	0.11	0.05	–
n = 56	0.001***	0.579	0.974	0.432	0.506	0.117	0.612	0.005***	0.04*	0.766	0.63	0.422	0.72	–

Statistically significant results are highlighted in bold.

*n* number of patients.

^a^S100B protein, ng/mL.

^b^White Blood Cell count per mm^3^.

^c^Lymphocyte count per mm^3^.

^d^Alanine Aminotransferase—IU/L.

^e^Creatinine—mg/dL.

^f^d-Dimer—ng/mL.

^g^Prothrombin—seconds.

^h^Ferritin—mg/L.

^i^Procalcitonin—ng/mL.

^j^C Reactive Protein—mg/dL.

^k^Patients’ age—years.

^l^Period from hospitalization to blood sample collection—days.

^m^Period from blood sample collection to hospital discharge—days.

^n^Covid score—%.

^+^*p* < 0.1; *p < 0.05; **p < 0.01; ***p < 0.001.

## Discussion

In this study, we investigated the levels of circulating S100B in serum of patients affected by SARS-CoV-2 virus at various stages of the disease. S100B serum concentrations resulted correlated with the severity of the disease, as indicated by clinical/laboratory parameters. The major part of data at present available on the S100B protein as a biomarker and pathogenic factor deals with disorders primarily related to the nervous system whereas disorders primarily related to other systems having been essentially disregarded^12^: thus, present results appear to enlarge the field of investigation on this protein and its potentials in Covid-19 and, more in general, infectious diseases. Indeed, the discrete distribution of S100B in definite extra-neural cell types offers the basis for a functional/pathogenic role of the protein in extra-neural tissues, which at present has not been exhaustively investigated.

This study was performed during the epidemic peak, with the advantage of collecting homogeneous data from the same outbreak but with all the restrictions present while managing the emergency, including the limits in sample size and in the number of additional clinical or laboratory parameters to be assessed. However, the acquired data were stressed by an accurate statistical analysis, strongly reporting a role for S100B in Covid-19.

The increased levels of S100B are reasonably related to inflammatory processes, as also supported by its significant correlation with CRP, which is known to be a recognized inflammatory hallmark^24^. S100B is known to participate in inflammatory processes^25,26^ which are also known to be raised during SARS-CoV-2 disease^19,21^. Interestingly, in this respect we observed that S100B levels are correlated with indicators of distress involving non-neural districts, such as ALT, d-Dimer, PCT, suggesting, in this case, a wider and systemic valence for S100B as a putative biomarker.

The source of increased serum S100B in SARS-CoV-2 patients remains to be identified. Since information indicating a prevalent involvement of the nervous system in pathogenic processes of SARS-CoV-2 at present is lacking^27^, it seems unlikely that in this case the protein is primarily released from this tissue, which at present is regarded to be the natural source of the protein in biological fluids in the major part of pathological conditions already known. Among the cell types which are known to express and putatively release the protein, adipocytes, dendritic and lymphoid cell types^28–31^ appear to be putative sources for serum S100B in this disease. Adipocytes are known to secrete inflammatory cytokines which play a recognized role in crosstalk with the immune system, which at present are known to be especially relevant in processes leading to obesity^32,33^. Since the role(s) of molecules secreted by this intriguing cell type, including S100B, is at present largely unknown, this finding might add a novel element deserving interest. In the case of dendritic and lymphoid cell types, their role in inflammatory processes is widely known^34,35^, so that the mechanistic involvement of S100B in their function would merely increase the breadth of knowledge in this respect. It is interesting to note that, under pulmonary inflammation, S100B has been reported to be upregulated in bronchiolar epithelial cells and airway dendritic cells^36,37^. Moreover, as shown in alveolar cell types, S100B can stimulate the secretion of pro-inflammatory cytokines, that are commonly involved in lung inflammation, following a similar process suspected to be present also in Covid-19^19–22,36,37^.

Additional studies will be needed in order to define the source of serum S100B in patients affected by SARS-CoV-2, but the finding of increased serum levels of the protein, correlated with the gravity of the disease and inflammatory processes, offers a novel biomarker potentially useful to monitor the disease. In addition, in the light of growing evidence candidating S100B as an active factor in inflammatory processes^12^, the present findings may even propose the protein as a therapeutic target to counteract the potent inflammatory processes characterizing this infectious disease.

In conclusion, increased serum levels of S100B correlate with the severity of Covid-19 and inflammatory processes, offering a novel biomarker potentially beneficial in monitoring the disease course and prognosis. In the light of growing evidence candidating S100B as an active factor in inflammatory processes driven by DAMP and RAGE, the present findings propose this protein as a severity marker and its cellular pathways as candidate targets to unravel pathogenetic mechanisms and counteract the potent inflammatory processes characterizing SARS-CoV-2 infection.

## Methods

### Dataset

74 serum samples from patients with confirmed SARS-CoV-2 infection hospitalized in an academic Covid hospital in Rome, Italy, were collected during the epidemic pick (from January 29th to May 6th, 2020). The inclusion criteria were Covid-19 diagnosis and age (≥ 18 y.o.), while exclusion criteria were concomitant or pre-existing neurological disorders, cardiovascular diseases, diabetes and cancer. According to WHO clinical criteria at admission time, patients were hospitalized in High (HIC) and Low Intensity Care (LIC) wards, respectively. Severity of Covid-19 at the time of blood sampling was quantified using a Covid-score, attributing a value ranging from 0 to 100%^4–7,9^. Main variables incorporated in the Covid-score included: older age, male sex, comorbidities, respiratory rate, oxygenation, radiographic severity, higher neutrophils, higher CRP and lower albumin at presentation, predicted critical care admission and mortality; in particular: age > 50, male, oxygen saturation < 93%, radiological severity score > 3, neutrophil count > 8.0 × 10^9^/L, CRP > 40 mg/L, albumin < 34 g/L, creatinine > 100 μmol/L, comorbidity and chronic lung disease, ALT > 40 IU/L; Creatinine > 100 μmol/L; D-dimer > 0.5 μg/L; Prothrombin-time > 16 s; Ferritin > 300 μg/L; Procalcitonin > 0.1 ng/mL.

Namely, patients recovered in HIC (n = 16) presented: severe pneumonia (fever or suspected respiratory infection, plus one of the following: respiratory rate > 30 breaths/min; severe respiratory distress or SpO2 ≤ 93% on room air); and LIC (n = 58) comprehended a group spanning from uncomplicated disease to pneumonia but without signs of severe pneumonia. In both groups, all patients received treatment in accordance with the guidelines available at the time of the study^1^. Time of hospitalization ranged from 7 to 85 days (average: 29.97). Table 1 shows patients’ characteristics together with data obtained on serum samples. Patients were balanced with respect to gender (female = 49%), their age ranging from 32 to 89 (median: 66.0). Serum samples from healthy subjects (n = 5) negative for SARS-CoV-2 detection by PCR and negative by serologic test were used as controls for the ELISA test.

### Ethics

The project protocol involved the rapid recruitment of patient-participants during the pandemic pick and no additional project-related procedures (we used only material from routine venipunctures). Anonymity was assured by the hospital privacy protocol and written consent obtained by each of recruited participants. The study was approved by the Sant’Andrea University Hospital Ethical Committee/Institutional Review Board (N. 5773/2020).

### Laboratory Tests

The serum S100B concentrations were measured by adapting the enzyme-linked immunosorbent assay (ELISA) kit following manufacturer protocols (S100B ELISA Kit, Abcam, England). Samples resulting below the detection limit of the test (LOD, equal to 0.1 ng/mL) were considered negative and assigned a value of 0 ng/mL. From the same serum, a panel of consolidated blood markers was also assessed, including White Blood Cell count (WBC), Lymphocytes (LYM), Alanine Aminotransferase (ALT), Creatinine (CRE), d-Dimer, Prothrombin (PT), Ferritin (FERR), Procalcitonin (PCT), C Reactive Protein (CRP).

### Statistical analysis

Continuous variables were S100B serum concentration (ng/mL), WBC (count per mm^3^), LYM (count per mm^3^), ALT (IU/L), CRE (mg/dL), d-Dimer (ng/mL), PT (seconds), FERR (ng/mL), PCT (pg/mL), CRP (mg/dL), period from hospitalization to blood sample collection (days), period from sample collection to hospital discharge (days), severity of the disease (Covid score), age (years). Categorical variables were gender (male = 0, female = 1), S100B protein in detectable amount (below LOD = 0 and over LOD = 1), hospitalization ward (LIC = 0 and HIC = 1). Relations between continuous variables were compared by Pearson’s correlation. Pearson’s χ^2^ test was used to compare the frequencies of the categorical variables and non parametric test Wilcox or Kruskal were used to compare the two groups under the non-normality assumption. Linear models (ANOVA, ANCOVA, and Linear Regression) were performed to evaluate the effect of variables observed on concentration of S100B. Whenever the test of homogeneity of variance was not satisfied, the ANOVA results were substituted by those from Welch ANOVA. Tukey test were performed to evaluate the difference of means between groups. If equal variance assumption is violated during the ANOVA process, pairwise comparison was based on the Games-Howell statistics. Stepwise regression was performed to obtain the model with the best predictors. All analyses were considered statistically significant at a p value of less than 0.05, if not differently indicated. All data were analyzed using the R environment for statistical computing (Version 4.0.1).

## Supplementary information

Supplementary information 1.

Supplementary information 2.
