# Supplementary material for: Serum S100B protein as a marker of severity in Covid-19 patients
Source: Sci Rep. 2020 Oct 29;10:18665. doi: 10.1038/s41598-020-75618-0 (PMC7596559; doi:10.1038/s41598-020-75618-0)
Supplement: Supplementary file 1 — Supplementary information 1. [file 41598_2020_75618_MOESM1_ESM.pdf]

Serum S100B protein as a marker of severity in Covid-19 patients

Aceti Antonio[1], Margarucci Lory Marika[2], Scaramucci Elena[1], Orsini Massimiliano[3], Salerno Gerardo[1], Di Sante Gabriele[4], Gianfranceschi Gianluca[2], Di Liddo Rosa[5], Valeriani Federica[2], RIA Francesco[4], Simmaco Maurizio[1], Parnigotto Pier Paolo[5], Vitali Matteo[6], Romano Spica Vincenzo[2*+], Michetti Fabrizio[4,7+]

1 Clinical Infectious Diseases, Sant'Andrea Hospital, Sapienza University of Rome, Via di Grottarossa 1035, 00189, Rome, Italy

2 Department of Movement, Human and Health Sciences, Laboratory of Epidemiology and Biotechnologies, University of Rome Foro Italico, Piazza Lauro De Bosis 6, 00135 Rome, Italy

3 Istituto Zooprofilattico Sperimentale delle Venezie, Viale dell'Università 10, 35020 Legnaro, Padua, Italy

4 Department of Neuroscience, Università Cattolica del Sacro Cuore, Largo francesco Vito 1, 00168 Rome, Italy

5 Department of Pharmaceutical and Pharmacological Sciences, University of Padua, Via 8 Febbraio, 2 - 35122 Padua, Italy

6 Department of Public Health and Infectious Diseases, Sapienza University of Rome, Piazzale Aldo Moro, 5, 00185 Rome, Italy

7 IRCCS San Raffaele Scientific Institute, Università Vita-Salute San Raffaele, Via Olgettina, 58, 20132 Milan, Italy

+These authors contributed equally to this article.

***Correspondence**: Vincenzo Romano Spica at the Department of Movement, Human and Health Sciences, University of Rome "Foro Italico", Piazza Lauro De Bosis 6, 00135 Rome, Italy Tel.: +39-06-36733-223; E-mail: vincenzo.romanospica@uniroma4.it

Supplementary material
(In support to data reported in figure 2 A)

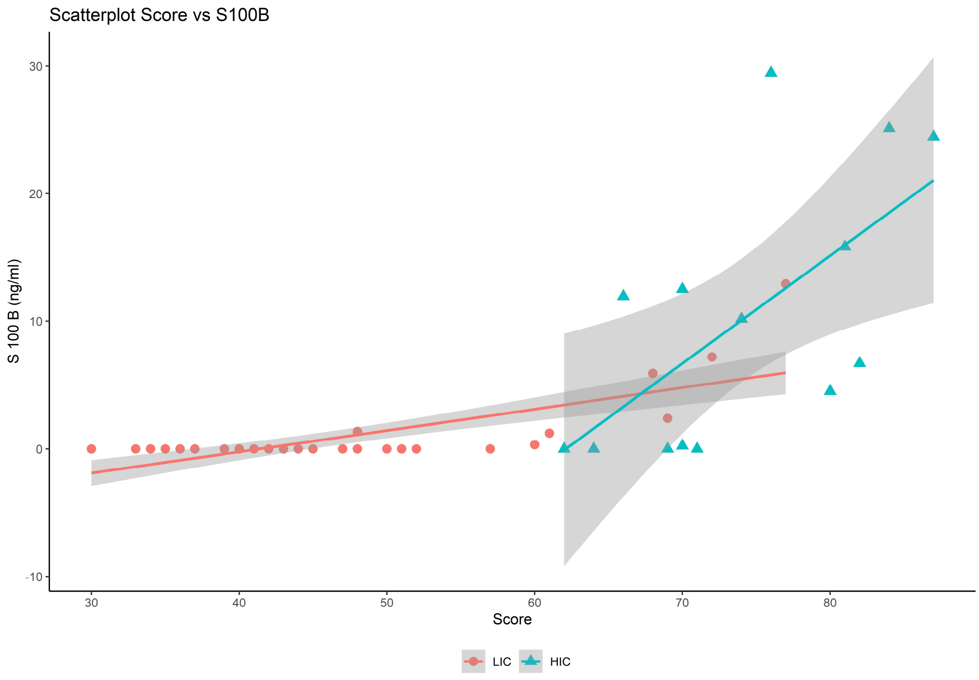

Figure S1: Linear regression curves in LIC and HIC

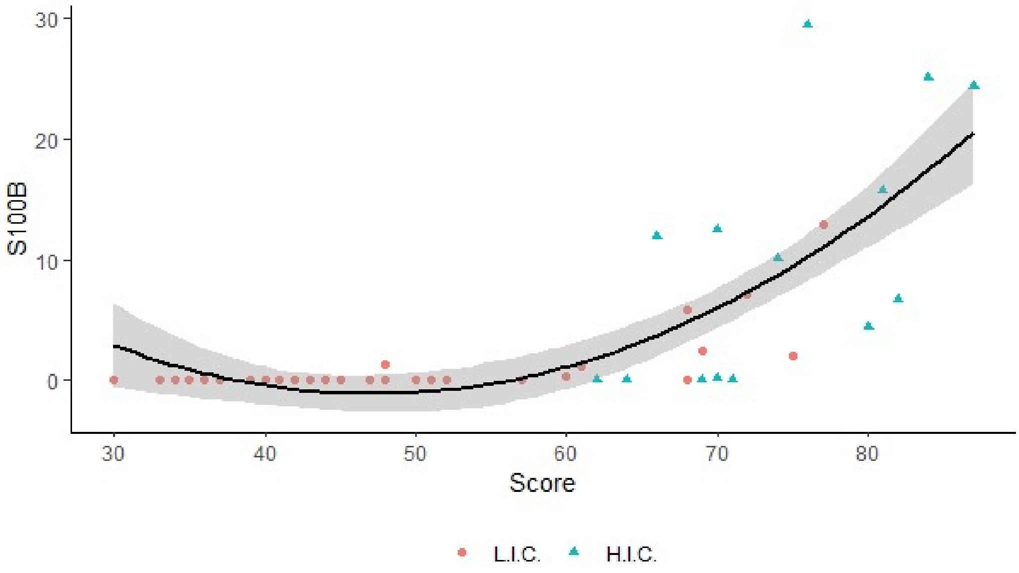

Figure S2: Quadratic regression
