# Supplementary material for: Serum S100B protein as a marker of severity in Covid-19 patients
Source: Sci Rep. 2020 Oct 29;10:18665. doi: 10.1038/s41598-020-75618-0 (PMC7596559; doi:10.1038/s41598-020-75618-0)
Supplement: Supplementary file 2 — Supplementary information 2. [file 41598_2020_75618_MOESM2_ESM.pdf]

Supplementary material

(In support to data reported in figure 2 A)

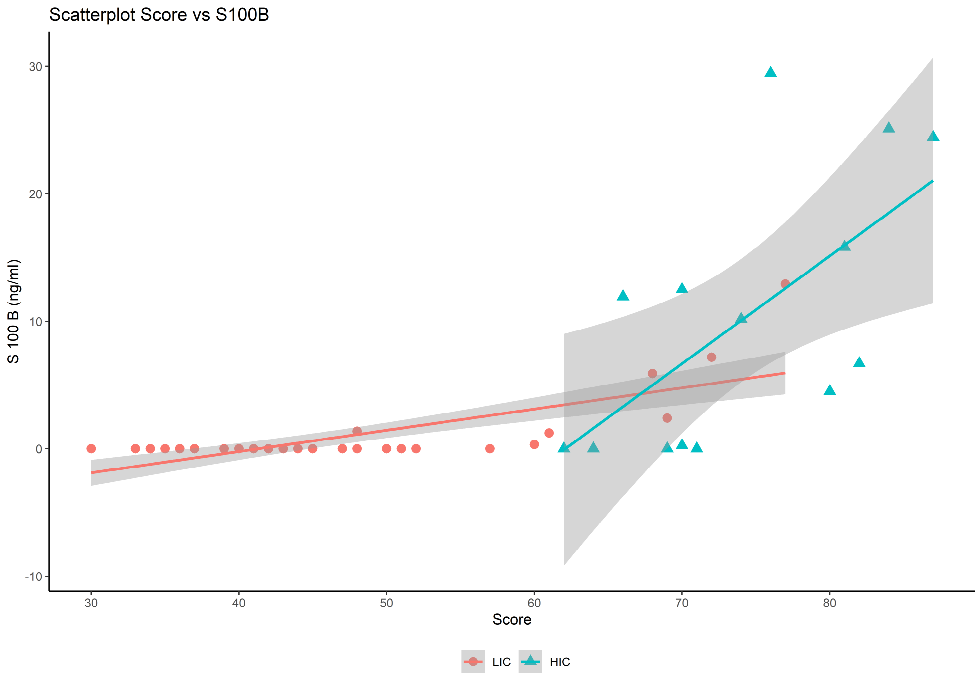

Figure S1: Linear regression curves in LIC and HIC

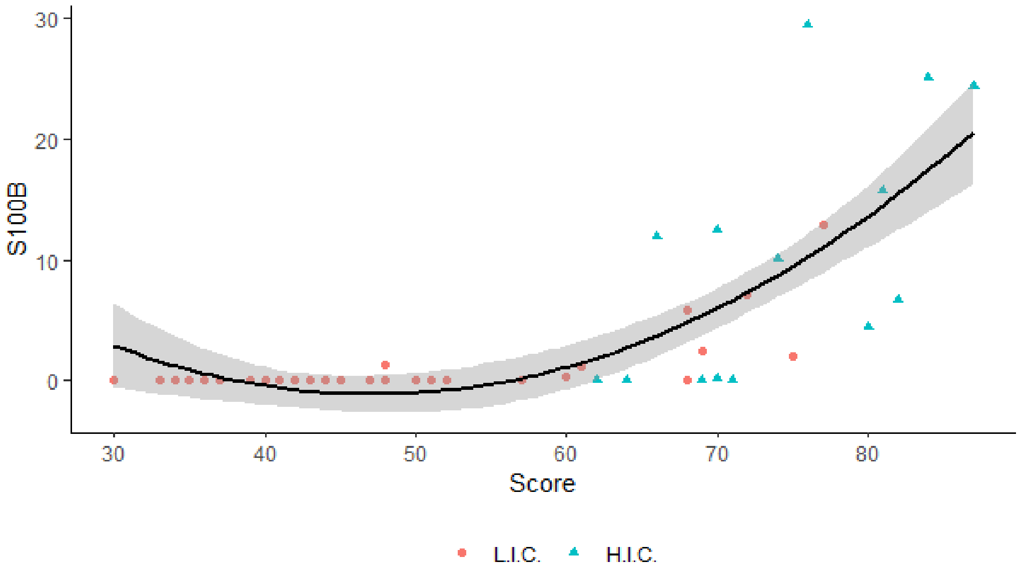

Figure S2: Quadratic regression
